# Brain Rest

**Published:** 1891-08

**Authors:** 


### Brain Rest.

Sir James Crichton Brown, in his course of lectures recently delivered upon the subject of "Brain Rest," said that ordinary sleep grows deeper for the first hour and a half and tben steadily diminishes until the slumberer awakens. Dr. Brown pleaded for eight hours for actively working brains, though ascetic notions have led many people to shorten the time, with the result that in certain cases it has been proved that the amount of sleep may be considerably reduced without injury. Literary men were apt to starve the brain in the matter of sleep, but some, nevertheless, had got on pretty well in spite of insomnia. Carlyle and Rossetti furnished instances. Dr. Brown quoted a letter from his friend Dr. Tyndall, who said: "For four weeks I have never had a single second of sleep, and during those nights I walked thousands of times round my room to no purpose. What astonishes me above all" (he adds) "is notwithstanding my nights' weariness my brain power does not appear to be sensibly impaired. After two or three hours' sleep I feel my brain as strong and clear as it ever was at any period of my life." It is, in Sir J.

Crichton Brown's opinion, impossible to doubt that nutrition and repair must have gone on in the brain during periods of sleeplessness. The brain in short must, as he expressed it, "have learnt the trick of the heart and gone to sleep during the beats, or it must have slept in centres which were not active at the same time.—*Exchange.*

